# A Fully Automated Online SPE-LC-MS/MS Method for the Determination of 10 Pharmaceuticals in Wastewater Samples

**DOI:** 10.3390/toxics10030103

**Published:** 2022-02-23

**Authors:** Masho Hilawie Belay, Ulrich Precht, Peter Mortensen, Emilio Marengo, Elisa Robotti

**Affiliations:** 1 Department of Science and Technological Innovation, University of Piemonte Orientale, Viale T. Michel 11, 15121 Alessandria, Italy; masho.belay@uniupo.it (M.H.B.); emilio.marengo@uniupo.it (E.M.); 2 Eurofins Environment Denmark A/S, Ladelundvej 85, 6600 Vejen, Denmark; ulrich.precht@weeco.com (U.P.); peterbondgaardmortensen@eurofins.dk (P.M.); 3 WEECO ApS, Vesterladenvej 21, 9310 Aalborg, Denmark

**Keywords:** antidepressant, antineoplastic agents, anticancer drugs, LC-MS/MS, method validation, online SPE, pharmaceuticals, renin inhibitor, wastewater

## Abstract

The increasing use of pharmaceuticals, their presence in the aquatic environment, and the associated toxic effects, have raised concerns in recent years. In this work, a new multi-residue analytical method was developed and validated for the determination of 10 pharmaceuticals in wastewaters using online solid-phase extraction (online SPE) and liquid chromatography-tandem mass spectrometry (LC-MS/MS). The compounds included in the method were antineoplastics (cabazitaxel, docetaxel, doxorubicin, etoposide, irinotecan, methotrexate, paclitaxel, and topotecan), renin inhibitors (aliskiren), and antidepressants (maprotiline). The method was developed through several experiments on four online SPE cartridges, three reversed phase chromatography columns, and four combinations of mobile phase components. Under optimal conditions, very low limits of detection (LODs) of 1.30 to 10.6 ng L^−1^ were obtained. The method was repeatable, with relative standard deviations (RSD, %) for intraday and interday precisions ranged from 1.6 to 7.8 and from 3.3 to 13.2, respectively. Recovery values ranged from 78.4 to 111.4%, indicating the reproducibility of the method. Matrix effects were mainly presented as signal suppression, with topotecan and doxorubicin being the two most affected compounds (31.0% signal suppression). The proposed method was successfully applied to hospital effluents, detecting methotrexate (4.7–9.3 ng L^−1^) and maprotiline (11.2–23.1 ng L^−1^). Due to the shorter overall run time of 15 min, including sample preparation, and reduced sample volume (0.9 mL), this on-line SPE-LC-MS/MS method was extremely convenient and efficient in comparison to the classical off-line SPE method. The proposed method was also highly sensitive and can be used for ultratrace quantification of the studied pharmaceuticals in wastewaters, providing useful data for effective environmental monitoring.

## 1. Introduction

For decades, several studies have evaluated the occurrence, effects, and risks of pharmaceutical compounds in the environment [1,2,3,4], with most of those studies concluding that pharmaceuticals can compromise water quality. The release of pharmaceuticals in effluents from manufacturing plants, hospitals, private households, as well as the inappropriate disposal of leftover medications, can contaminate surface water, ground water, and, eventually, drinking water [5]. Pharmaceuticals are commonly found in aquatic compartments at very low concentrations ranging from ultratrace (ng L^−1^) to trace (µg L^−1^) levels, which are currently regarded as potential hazard for a variety of living organisms, including humans [6,7]. Moreover, recent studies have shown that pharmaceuticals’ levels in urban wastewaters are rising due to population aging and the increase in population density [8].

The removal efficiency of pharmaceuticals greatly varies among different wastewater treatment systems, and a significant amount of the parent drugs and their transformation products may pass through and enter the aquatic environment [4,9,10,11] and wetlands [12]. The occurrence of pharmaceuticals in the environment is affected by their overall consumption and their fate in the environment. As a result, pharmaceuticals with larger consumption rates have been commonly detected in wastewater, surface water, and even in drinking water [13]. Nowadays, thanks to the advancements in analytical instruments and the significant improvement in sensitivity of analytical methods, ultratrace analysis of a large group of pharmaceutical compounds in various aquatic matrices is becoming a common practice.

The continuous rise of cancer cases has led to the increased use of anticancer drugs and a further increase in their use in the years to come can be foreseen [14,15]. Anticancer drugs (also known as antineoplastics) are generally administered in hospitals and, thus, hospital wastewater is considered as the one of the various routes through which those compounds reach the aquatic environment [16]. In addition to this, domestic wastewater and, eventually, wastewater treatment plants (WWTPs) are key pathways since most cancer patients leave the hospital after receiving treatments [16]. According to the World Health Organization (WHO), anticancer drugs are classified under the class L—antineoplastic and immunomodulating agents [17], subclass L01X—other antineoplastic agents. Although the overall consumption varies by country, the anticancer drugs broadly used in chemotherapy include cyclophosphamide, ifosfamide, 5-fluorouracil, methotrexate, gemcitabine, azathioprine, doxorubicin, tamoxifen, etoposide, vincristine, chlorambucil, docetaxel, irinotecan, and paclitaxel [17,18,19]. Studies have indicated that anticancer drugs could exert cytotoxic, genotoxic, mutagenic, carcinogenic, or teratogenic effects on aquatic species [17,20]. Furthermore, other studies have found that antineoplastics have low degradability by conventional wastewater treatments [21,22]. As a result, anticancer drugs, their metabolites, and transformation products have been identified as potential emerging contaminants of concern (CECs), requiring substantial research into their incidence and concentrations in aquatic ecosystems. In agreement with this, a growing number of publications and official reports have been released focusing on the occurrence and potential effects of antineoplastics in wastewater effluents and influents [23,24,25,26,27], while some studies have also measured them in surface and ground waters [28,29,30].

In this study, eight antineoplastics, namely methotrexate (MTX), docetaxel (DTX), etoposide (ETP), irinotecan (IRI), topotecan (TOP), cabazitaxel (CTX), paclitaxel (PTX), doxorubicin (DOX), were selected. In addition, maprotiline (MAP)—an antidepressant, and aliskiren (ALK)—an antihypertensive, were included making up a total of 10 pharmaceuticals. The compounds were chosen based on consumption data in Europe, excretion rate of the compound’s unaltered form, and frequency of detection (when available) in effluent and influent WWTP samples [8,13,17,18,31,32]. Excretion of the unmetabolized forms were significantly large for the selected compounds. For example, 60–95% MTX; 25–45% ETP and TOP; 5–15% PAC, DTX, CTX and DOX; 15–25% IRI; up to 80% ALK [18,32,33]. Furthermore, most of the selected target analytes were reported to have been detected and quantified in surface waters, and urban and hospital wastewaters (influents and effluents) in fairly low to medium concentrations, including 1.6–300 ng L^−1^ MTX [19,24,27,34], 18.5–100 ng L^−1^ PTX [3,27], 9.0–10 ng L^−1^ MAP [35,36], 2.5–2.7 ng L^−1^ DOX [27], 0.4–60 ng L^−1^ IRI [25,27,30,34], 3.4–15 ng L^−1^ ETP [25], 0.4–1900 ng L^−1^ ALK [37], 97.7–175.1 ng L^−1^ DTX [3]. Once they enter the aquatic environment, these compounds can produce transformation products whose effects are merely known [38,39,40,41]. Despite their extensive usage and the relatively high excretion rates in their unmetabolized forms, CTX and TOP have not been previously detected in environmental samples. In general, information on the occurrence of all the selected target compounds in the environment are still scarce and a rapid, sensitive, and reproducible multi-residue analytical method is required to be able to quantify them at low concentrations.

There are several techniques employed for the extraction and preconcentration of pharmaceuticals from aqueous samples, including pressurized liquid extraction and ultrasound-assisted extraction [42], dispersive liquid–liquid microextraction [43], stir bar sorptive extraction [44], and QuEChERS [45]. However, solid-phase extraction (SPE) has been shown to be the most effective extraction approach for pharmaceutical compounds in water [46,47], with numerous SPE variants available, including offline, online, cartridges, and disks [48]. Conventional off-line SPE techniques often require large quantities of samples and/or high concentrations of analytes, which can be quite different from real environmental conditions [49]. Automated procedures and devices that integrate extraction, separation, and detection phases have gained popularity over the last decade due to their multiple benefits, which include minimal sample handling, small sample volume, and reduced solvent use [50]. Therefore, automation of the SPE system, online with the LC-MS/MS system, as is the case in this work, greatly decreases the overall run time and enables the treatment of more samples.

The most frequently used analytical techniques to determine trace levels of pharmaceuticals in water are high performance liquid chromatography coupled with ultraviolet detection (HPLC-UV) [51] or mass spectrometry (HPLC-MS) [5], as well as gas chromatography-mass spectrometry (GC-MS) [52]. Due to the polar, non-volatile, and thermally unstable nature of pharmaceuticals, LC-MS and LC-MS/MS are frequently used to analyze them in aqueous samples [46]. The popularity of LC-MS techniques is attributed to a variety of advantages, including the capacity to offer structural information, the speed and convenience of analysis, the potential to analyze multicomponent mixtures, and the ability to provide accurate quantification [45]. Tandem MS (MS/MS) systems have several advantages over single quadrupole (MS) systems, as detailed elsewhere, e.g., [53,54]. MS/MS systems exhibit higher selectivity, which means they require less HPLC separation due to reduced interference from co-eluting compounds and the matrix. Additionally, MS/MS offers a higher signal-to-noise ratio (S/N), allowing for lower quantitation limits. Furthermore, Collision Induced Dissociation (CID) of the precursor masses in MS/MS results in the formation of product ions, which provide additional structural information. On top of that, the Multiple Reaction Monitoring (MRM) mode in MS/MS provides more reliable identification of detected analytes than the Selected Ion Monitoring (SIM) in MS systems. Other advantages of MS/MS include a wider linear range, improved accuracy and reproducibility, particularly at low concentrations.

It is important to note that developing a sensitive multi-residue method able to determine many pharmaceuticals belonging to diverse classes remains to be an analytical challenge. Moreover, because of the large number of pharmaceuticals currently used, targeted methods have been developed to prioritize those compounds highly expected to be detected in the environment. For this reason, efforts to ascertain several other pharmaceutical compounds, which are likely to pose a risk to the aquatic environment, are essential. When sufficient data on the environmental concentrations of these rather less studied compounds is documented, it could potentially attract more studies to be carried out focusing on the fate, effects and risks to aquatic organisms and humans.

Based on the considerations discussed above, we developed and validated an online SPE-LC-MS/MS method for the determination of ten pharmaceutical compounds in wastewaters. Details on the selected target drugs are given in Table S1 (Supplementary Materials). Along with the ten compounds described above, we originally attempted to analyze other anticancer drugs simultaneously using the same method but were unable to do so due to their vastly differing physico-chemical characteristics. The compounds were bortezomib, gemcitabine, mitomycin, 5-fluorouracil, vinblastine, and vincristine. For the extraction and analysis of the ten target analytes, we utilized online solid-phase extraction (SPE) combined with high performance liquid chromatography-tandem mass spectrometry (HPLC-MS/MS). As compared to offline SPE, online SPE was the preferred sample preparation method since it allowed the development of a rapid analytical method with improved analytical results, thus reducing the overall analysis time and organic solvent consumption.

## 2. Materials and Methods

### 2.1. Chemicals and Reagents

All solvents were of LC-MS grade, and all chemicals were of analytical reagent grade. Acetonitrile (Honeywell Riedel-de Haen™, Chromasolv^TM^ Plus, for HPLC, ≥99.9%) and formic acid (Chemsolute^®^, ACS, 99–100%) were obtained from Th. Geyer GmbH (Renningen, Germany). Methanol (LiChrosolv^®^, for HPLC, ≥99.8%), water (LiChrosolv^®^, LC-MS Grade), and hydrochloric acid (Emsure^®^, ACS, 37%) were purchased from Merck (Darmstadt, Germany). Ammonium formate buffer (5 mol L^−1^) was purchased from Agilent Technologies (Waldbronn, Germany). Ultrapure water was generated using a Millipore Milli-Q^®^ Gradient water purification system and had a resistance of 18.2 MΩ cm^−1^ (at 25 °C) and TOC value below 5 ppb.

Doxorubicin hydrochloride (CAS No. 25316-40-9; purity 98–102%), etoposide (CAS No. 33419-42-0; 98–105%), topotecan hydrochloride hydrate (CAS No. 123948-87-8; ≥98%), paclitaxel (CAS No. 33069-62-4; ≥95%), docetaxel (CAS No. 148408-66-6; ≥97%), methotrexate (CAS No. 59-05-2; ≥98%), and irinotecan hydrochloride (CAS No. 136572-09-3; ≥97%) were purchased from Sigma-Aldrich (Milan, Italy). Cabazitaxel (CAS No. 183133-96-2; ≥95%), maprotiline hydrochloride (CAS No. 10347-81-6; >99%), and aliskiren (CAS No. 173334-57-1; ≥98%) were from Merck (Darmstadt, Germany). Atrazine-d_5_ (100 mg L^−1^) was purchased from Dr. Ehrenstorfer GmbH (Augsburg, Germany) and used as the internal standard (IS). Stock solutions (1000 µg mL^−1^) of each analyte were prepared in methanol and stored in amber glass vials. Furthermore, 25 µg mL^−1^ standard mixture (mix) of all the ten analytes was prepared in methanol. All vials were stored in a dark standard-only freezer at −20 °C. For method optimization, working solutions (1 µg mL^−1^) were made by diluting the stock solutions in ultrapure water. Similarly, calibration standards were prepared by appropriate dilution of the mix in methanol/water (10:90, *v*/*v*).

### 2.2. Safety

To guarantee the best possible protection for workers and the workplace, the target compounds used in this method were treated as potential health hazards and handled with extreme caution in line with their individual safety data sheets. All stock solutions were made in a biological safety hood with laminar airflow and absorbent paper on the work surfaces. All disposable materials that came in contact with the compounds under investigation were discarded as hazardous waste.

### 2.3. Instrumentation

All analyses were performed by Agilent 1260 Infinity High-Performance Liquid Chromatography (HPLC) system (Agilent Technologies, Santa Clara, CA, USA) equipped with a quaternary pump (G1311C) used for sample loading into the SPE and a binary pump (G1312B) for elution of the analytes from the SPE cartridge and subsequent separation in the analytical column. The system consisted of an Agilent 1260 Infinity Standard Autosampler with a 900-µL loop (G1329B ASL), Agilent 1260 Infinity Thermostated Column Compartment (G1316A TCC), and Agilent Valve Drive (G1170A) with Agilent 1200 series 2-position/6-port valve (G1158A). The HPLC system was interfaced with an Agilent 6460 Triple Quadrupole Mass Spectrometer (G6460C TQ) which included an electrospray ionization (ESI) source with Agilent’s Jet Stream technology. During the optimization, four online SPE cartridges were tested, namely, Hypersil GOLD^TM^ aQ online column (2.1 × 20 mm, 12 µm; Thermo Fisher, Waltham, MA, USA), HyperSep^TM^ Hypercarb (2.1 × 20 mm, 7.0 µm; Thermo Fisher, Waltham, MA, USA), PLRP-s (2.1 × 12.5 mm, 15–20 µm; Agilent Technologies, Santa Clara, CA, USA), and Oasis HLB (2.1 × 20 mm, 5.0 µm; Waters, Milford, MA, USA). Moreover, three different analytical columns were tested: Eclipse Plus C18 column (150 × 2.1 mm, 3.5 µm; Agilent Technologies, Santa Clara, CA, USA), Kinetex C18 column (2.1 × 150 mm, 2.6 µm; Phenomenex, Aschaffenburg, Germany), and Luna Omega Polar C18 (150 × 2.1 mm, 3.0 µm; Phenomenex, Aschaffenburg, Germany). The final optimized method utilized the Hypersil GOLD^TM^ aQ online SPE column from Thermo Fisher and the Kinetex C18 separation column from Phenomenex. All qualitative and quantitative data were evaluated employing the Agilent MassHunter Workstation software.

### 2.4. Sample Collection and Preparation

First, the method was optimized using ultrapure water and wastewater influent obtained from Vejen (Denmark). The optimized method was subsequently employed to the analysis of six effluents of hospital wastewaters collected from Aalborg in Denmark (coded as A1 and A2) and Valencia in Spain (coded as V1, V2, V3, and V4).

Sample collection bottles were 500 mL capacity amber glass bottles with Teflon-lined caps which were thoroughly cleaned as follows: rinsing three times with tap water, three times with organic-free water, twice with washing acetone, once with special UV-grade acetone, twice with pesticide grade hexane and dry (uncapped) in a hot air oven at 360 °C for 24 h. All samples were collected using the pre-cleaned amber glass bottles. During sampling, the bottles were first rinsed twice with roughly 100 mL of the sample before filling them up. The effluents from Denmark were collected from two different sampling stations (A1 and A2) at a hospital in the region of North Jutland. Similarly, effluents from Spain were obtained from four sampling stations (V1, V2, V3 and V4). In all cases, grab samples of 500 mL were collected using small centrifugal pumps. All collected samples were immediately transferred into an ice-cooled container and delivered to the lab in chilled conditions. Upon arrival in the lab, water samples were acidified with HCl to pH 2 to minimize microbial degradation before being filtered first using Whatman 1.6 µm fiberglass filters and then 0.45 µm nylon membrane filters. The original pH was restored using NaOH solution and samples were always extracted within 24 h of collection. When this was not practicable, samples were kept frozen at −20 °C until analysis.

### 2.5. Optimization of LC-MS Conditions

To achieve the best online SPE-LC-MS performance for individual analytes, a series of experiments were performed to: optimize compound-dependent MS parameters and establish the MRM method; select the best online SPE column from 4 different variants that had improved overall recoveries; determine the appropriate online SPE loading solution; select the best analytical column among the 3 columns tested; and determine the appropriate HPLC mobile phase composition and optimize the gradient conditions. The results obtained at each stage are summarized under Section 3.1 and Section 3.2.

#### 2.5.1. Optimization of the MRM Method

To assess the chromatographic nature of the target compounds, a scouting reversed-phase LC-MS analysis was initially performed by analyzing the standards of each compound (1.0 µg mL^−1^) using an Eclipse Plus C18 column (150 × 2.1 mm, 3.5 µm), and a generic gradient of the mobile phases water/acetonitrile both containing 0.1% (*v*/*v*) formic acid. This preliminary assessment was conducted by applying an MRM method, created based on precursor ion and product ion transitions available in the literature [25,30,38,39,55]. The identification of all 10 target analytes was confirmed at this point, however several of them had extremely low sensitivities. As a result, the actual step-by-step method development and optimization was carried out as described in the subsequent sections.

Following the scouting analysis, automated optimization, using the Agilent MassHunter Optimizer software (version B.09.00), of the compound-dependent parameters for the MRM method was performed using 50 ng mL^−1^ standard solutions of each compound. The Optimizer automatically executed four acquisitions for each target ion: (i) MS^2^ SIM scan acquired data to optimize the fragmentor voltage for each precursor ion tuning from 100 to 200 V with a step of 5 V, (ii) Product Ion scan identified product ions for each precursor ion, (iii) MRM scan optimized the collision energy (CE) tuning from 5 to 50 V with a step of 2 V by defining an MRM mode on the Product Ions found in (ii), and (iv) Product Ion scan validated the optimal CE and masses using a smaller scan range for Product Ions. Additional details can be found in the Supplementary Materials, Optimizer S1 and Figure S1. The goal of this optimization procedure was to maximize precursor ion transmission by reducing collision-induced dissociation (CID) with fragmentor voltage, which was crucial for achieving the highest possible sensitivity for each target analyte. Moreover, product ion signals were maximized with CE, resulting in enhanced detection and quantification of the compounds. For each analyte, two of the most intense precursor ion/product ion transitions were identified and the one with the greater response was chosen to be the quantifier ion, while the other was the qualifier ion.

#### 2.5.2. Selection of Online SPE Cartridges

To ensure an effective and reproducible sample pre-treatment procedure, online SPE optimization experiments focusing on the type of SPE sorbent were performed. As described under Section 2.3, the online SPE cartridges tested were the polymeric PLRP-s cartridge from Agilent, the Hypersil Hypercarb and Hypersil GOLD aQ cartridges from Thermo Fisher, and the Oasis HLB cartridge from Waters. The loading solution was composed of 0.1% (*v*/*v*) formic acid in water and methanol (gradient given in Table 1).

**Figure 1:**
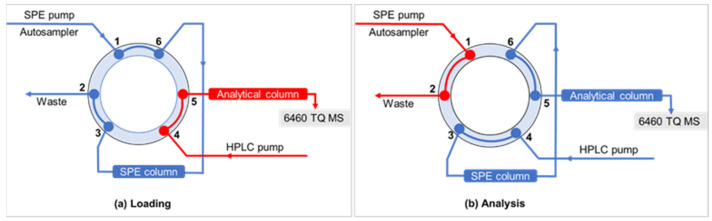
Configuration of the online SPE and the six-port switching valve.

The automated SPE-LC-MS/MS procedure began with loading (Figure 1a) of 900 µL water sample onto the SPE cartridge for 1.1 min at a flow rate of 1 mL min^−1^. While the sample matrix flowed to waste, the analytes were retained and concentrated on the SPE column. The analytical column was simultaneously equilibrated by the HPLC pump Then, by activating the divert valve of the column switching array (Figure 1b), the concentrated sample was flashed out of the SPE column and passed to the analytical column, where the analytes were separated and transferred for detection to the mass spectrometry equipment. At 10.0 min, the switching valve was returned to the load position to re-equilibrate both the SPE and the chromatographic column for 5.0 min. The detailed SPE conditions are presented in Table 1 along with the LC gradient program.

#### 2.5.3. Optimization of LC-Dependent Conditions

To optimize LC-dependent conditions, we tested different mobile phase compositions focusing on the type of organic phases and the modifiers. Methanol and acetonitrile were tested as the organic phases with or without formic acid (0.1%, *v*/*v*) as additive. In addition, the aqueous phase modifiers, formic acid (0.1%, *v*/*v*) and ammonium formate (5 mM, pH 3), were evaluated. Furthermore, the chromatographic separation of the analytes was evaluated using three different analytical columns: Agilent’s Eclipse Plus C18, and Phenomenex’s Kinetex C18 and Luna Omega Polar C18. After selecting the optimal column and mobile phase, we modified the elution gradient, column temperature, and flow rate.

Table 1 presents the online SPE and HPLC conditions for the optimized method. As a result of better analyte recoveries and good peak shapes achieved for most compounds (i.e., relatively narrower, and symmetrical peaks), the Hypersil GOLD aQ online column combined with the Kinetex C18 analytical column were selected respectively for preconcentration and separation of all the target analytes. The chromatographic separation was accomplished using a binary mobile phase system consisting of water (C in Table 1) and acetonitrile (D in Table 1) both containing 0.1% (*v*/*v*) formic acid. The column and autosampler temperatures were set at 40 °C and 4 °C, respectively. The mobile phase flow rate was 0.4 mL min^−1^ and the injection volume was 20 µL. The elution began at 5% D, held for 1.15 min before increasing to 100% D in 5.0 min and held for another 3.0 min, and finally returned to initial conditions in 2.0 min. The column was equilibrated for 5.0 min at the initial elution conditions before the next injection. The online SPE procedure was fully automated, and the total chromatographic run was 15 min. In order to eliminate/minimize carryover effects, a washing step for the syringe and the injection valve was programmed before each injection, first with 0.1% (*v*/*v*) formic acid in acetonitrile and then with 0.1% (*v*/*v*) formic acid in ultrapure water. Furthermore, after every eight samples, a blank control water sample was run through all steps in processing to check for target analyte carryover. Cross-contamination was controlled by evaluating peak areas of each target compound in the between-run blank relative to the neat blank run at the start of the analysis sequence and setting a 5% threshold. Blank injections were repeated (in some cases up to 4 times) until peaks for all compounds fell under this threshold.

The mass spectral data were acquired using the ESI source parameters presented in Table S2 (Supplementary Materials). Dynamic multiple reaction monitoring (dMRM) was used to monitor two specific transitions for each analyte over a delta retention period of 1-min with a dwell time of 150 ms. To confirm the presence of an analyte in a sample, the criteria of the SRM ratio between the qualifier and quantifier transition as suggested by European Commission Decision 2002/657/CE [56] and comparison of the retention time with that of the authentic standard were adopted. Quantification was achieved with calibration curves established using the analyte peak area of quantifier ions and the standard concentrations.

#### 2.5.4. Method Validation

The performance of the optimized method was validated using ultrapure water and a wastewater influent spiked with known concentrations of the target compounds. The parameters included in the validation study, evaluated according to the ISO/IEC 17025 guideline [57], were: selectivity, sensitivity, linearity, precision (interday and intraday repeatability), recovery and matrix effect. All LC-MS/MS analyses were performed in triplicate unless otherwise indicated, and the data were expressed as mean values.

Selectivity was evaluated by comparing the MS chromatograms of a blank sample and a blank sample spiked with 10 analytes and the IS (atrazine-d_5_). With the final optimized LC conditions and MRM transitions used, all the analytes were resolved without interference from the matrix at the respective retention times and both MRM transitions of the analytes.

Calibration standard mixtures were prepared at eleven concentration levels ranging from 1.0 to 1.0 × 10^3^ ng L^−1^ (LOQ, 1.00, 2.50, 5.00, 10.0, 25.0, 50.0, 100, 250, 500, and 1.00 × 10^3^ ng L^−1^) with the addition of 250 ng L^−1^ IS and 0.1% (*v*/*v*) formic acid, which were then analyzed in three replicates, applying a randomized injection order, using the optimized online SPE-LC-MS/MS method under dMRM. For each analyte, linear calibration curves were constructed by correlating the analyte peak area of the quantifier transition (y-axis) to the analyte standard concentration (x-axis). The Hubaux-Vos method [58] was used to evaluate linearity, lack-of-fit, and residuals of the calibration curves, as well as to calculate LOD and LOQ values. In contrast to the most commonly used ordinary least squares regression methodology, which considers errors only on the y-axis, the Hubaux-Vos method analyzes errors on both axes. Significant x-errors can be generated by factors such as temperature, reagent and solvent purity, and processes such as weighing and dilution. By accounting for the contributions of such variations, more practical estimates can be obtained applying the method used here.

The method’s sensitivity was evaluated by measuring the response of target analytes in successive dilutions of a concentrated working solution until the signal-to-noise ratio (S/N) of individual analytes reached a value >3 for the limit of detection (LOD) and >10 for the limit of quantification (LOQ).

Method precision was evaluated by analyzing spiked QC samples at three concentration levels for each analyte: QC_L_ (the LOQ), QC_M_ (100 ng L^−1^), and QC_H_ (800 ng L^−1^). A measure of 250 ng L^−1^ IS was added into all QC samples. Precision was expressed as the relative standard deviation, RSD %, for the analysis of five replicates of the QC samples in a single day (interday precision) and five replicates of each QC sample analyzed on three consecutive days (intraday precision).

Matrix effect (*ME*) was evaluated using wastewater influent samples at three QC levels by comparing the analyte mean peak areas of standards prepared in solvent (*A_solvent_*) with those of spiked wastewater influent samples (*A_spike_*) after correcting for the peak areas of the target compound in the unspiked wastewater influent (*A_blank_*). Three independent replicates were analyzed at each QC level and were reported as percentages (*ME* %) calculated using the following Formula (1):

(1) $$ME\;\left(\%\right)=\frac{A_{spike}-A_{blank}}{A_{solvent}}\times 100$$

Recoveries of analytes from real water matrices were also determined at three QC levels, following the same procedure as matrix effects. *Recoveries* (%) were calculated using the following Equation (2):

(2) $$Recovery\;\left(\%\right)=\frac{C_{spike}-C_{blank}}{C_{actual}}\times 100$$

where *C_spike_* was the measured concentration of the analyte in the spiked wastewater matrix, *C_blank_* was the original concentration of the analyte in the wastewater matrix, and *C_actual_* was the actual concentration spiked in the wastewater matrix.

## 3. Results and Discussion

### 3.1. Optimization of the HPLC-MS/MS

A sensitive and reproducible online SPE-HPLC-MS/MS method for the analysis of 10 pharmaceuticals of emerging concern was developed to meet the ever-increasing demand for the large-scale determination of target drugs in environmental water samples. The method was optimized by fine-tuning several critical parameters that were directly related to the extraction, chromatographic and mass spectrometric behaviors of the target drugs. Table 2 presents the formula of the 10 pharmaceuticals and the IS, the retention times, and the optimized LC-MS parameters.

**Figure 2:**
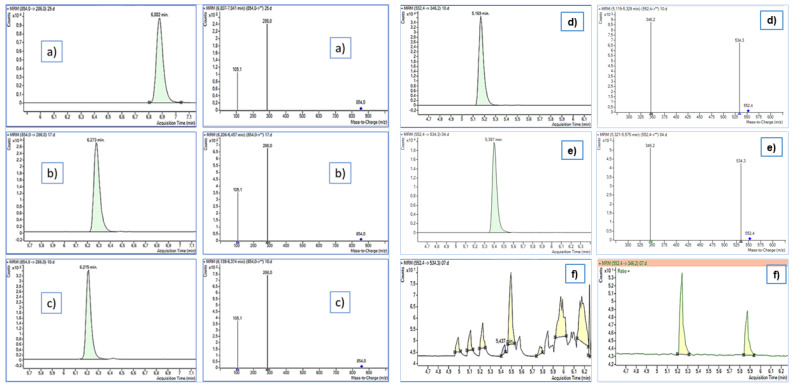
Retention differences for paclitaxel and aliskiren by the three analytical columns. PTX by Kinetex (**a**), Eclipse (**b**), and Luna Omega (**c**); ALK by Kinetex (**d**), Eclipse (**e**), and Luna Omega (**f**).

The pharmaceutical compounds targeted in this study consisted of eight anticancer drugs, one antidepressant, and one antihypertensive. According to recent studies, C18-based analytical columns are the most suitable and often employed for the analysis of a similar group of compounds in water [14,30,32]. In this work, we tested three reversed-phase C18 analytical columns of which one column had additional polar functionality. A 15-min chromatographic run was established for each column. The performance of Luna Omega Polar C18 towards the nonpolar high-molecular-weight compounds was extremely poor. The Kinetex C18 and Eclipse Plus C18 columns, on the other hand, enabled the separation of all 10 compounds with better sensitivity and improved peak shapes. In general, the Kinetex C18 achieved higher sensitivity for most compounds, which also provided peaks with improved efficiency and symmetry (asymmetry factor ranged from 0.95 to 1.61, Table S3). As an example, Figure 2 depicts the performance of the three columns, with PTX indicating a condition in which the columns had comparable performance and ALK demonstrating a clear difference in its retention by the columns and abundance of the product ions in the MS. The enhanced chromatographic peak profiles of the target compounds obtained using the Kinetex C18 stationary phase could be partially explained by the higher peak capacities and greater sensitivities of the core-shell technology compared to the fully porous columns since this 2.6 µm particle size column performs like a fully porous sub-2 µm columns [59,60]. Therefore, the Kinetex C18 column was selected for chromatographic separation of the target pharmaceutical compounds in the final optimized method.

The mobile phase composition and chemical changes in the solute can have an impact on the processes occurring within the column. Changes in organic solvent and additive concentrations, pH, and other variables such as ionic strength can all affect peak profiles. We investigated a series of mobile phase compositions and the results showed that adding low concentrations (0.1%, *v*/*v*) of formic acid both to the aqueous and organic phases greatly improved peak shape, detector signal intensity and S/N ratio of the precursor ion detected under SIM mode. When ammonium formate was used as an additive, distorted peaks were obtained in addition to poor ionization and co-elution of the target compounds. Taking into account all these results, a mobile phase system composed of water and acetonitrile, both containing 0.1% (*v*/*v*) formic acid, provided better peak profiles (peak shape, sensitivity, resolution) for the majority of the analytes. The addition of formic acid was necessary to boost ionization in positive ESI mode and improve peak shapes.

### 3.2. Optimization of the Online SPE

**Figure 3:**
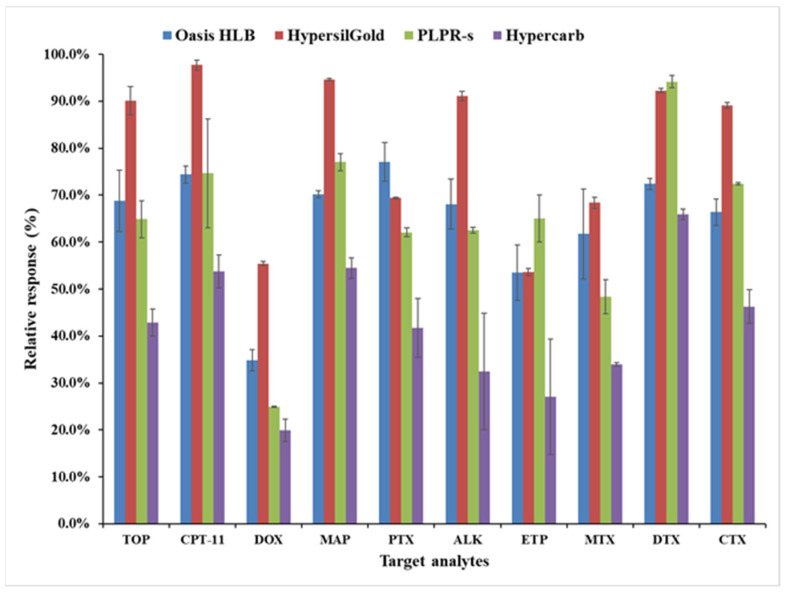
Comparison of the recoveries (relative peak area responses) obtained with four online SPE cartridges (analyte concentration 1.0 µg L^−1^, sample volume 500 µL, *n* = 3 replicates).

Following the LC-MS optimization, a series of experiments were performed to select the best online SPE cartridge for the extraction of the target analytes. Choosing an online SPE cartridge capable of providing high recoveries for all target analytes is a crucial step in developing a reproducible method. To this effect, four online SPE cartridges were evaluated: Hypersil GOLD aQ, Hypersil Hypercarb, PLRP-s, and Oasis HLB. Samples were prepared in three replicates by spiking ultrapure water with a mix of the target compounds at 1.0 µg L^−1^ levels. For each analyte, the extraction efficiency of each online SPE cartridge was calculated as percentages of the peak areas obtained for the online SPE analysis and a direct chromatographic injection of an equivalent amount of the standard mixtures. The recoveries calculated as the relative response of peak areas obtained with all four cartridges are shown in Figure 3 (numerical values of recoveries are also given in Table S4).

The selection of the SPE sorbent depends essentially on the physico-chemical characteristics of the target analytes and the nature of the matrix. For most analytes, the Hypersil GOLD aQ (C18, Octadecyl) and PLRP-s (a crosslinked styrene-divinylbenzene polymer) exhibited good recoveries with acceptable repeatability. The Oasis HLB (a macroporous copolymer of divinylbenzene and n-vinylpyrrolidone) on the other hand, produced lower recoveries and repeatability for some compounds. The results obtained using PLRP-s and Oasis HLB partly agree with a previous report [30], in which the PLRP-s showed better efficiencies for irinotecan, while Oasis HLB provided good recoveries for methotrexate, etoposide, doxorubicin, and paclitaxel. In the present study, the performance of the Hypercarb online SPE cartridge was characterized by low recoveries and repeatability. This cartridge contained porous graphitic carbon (PGC) suitable for the retention of highly polar compounds, and the poor recoveries obtained in this study could be due to the low polarities of the target compounds. In the final optimized method, the Hypersil GOLD aQ online column was selected for extraction of all target analytes since it showed better retention and less peak broadening (narrower and symmetrical peaks, Table S3) for most of the analytes. In the literature, the Hypersil GOLD aQ online column was found to have good recoveries for the extraction of synthetic and natural estrogens from river water and wastewater [61]. Moreover, the results were reproducible, as evidenced by the very modest error bars of triplicate analyses reported in Figure 3. In general, the SPE method not only improved the analytical results but also reduced the analysis time and the solvent consumption. The greater peak broadening seen for the other SPE columns might be attributed to the incompatibility of their stationary phases with that of the analytical column [26,62].

### 3.3. Method Validation

**Figure 4:**
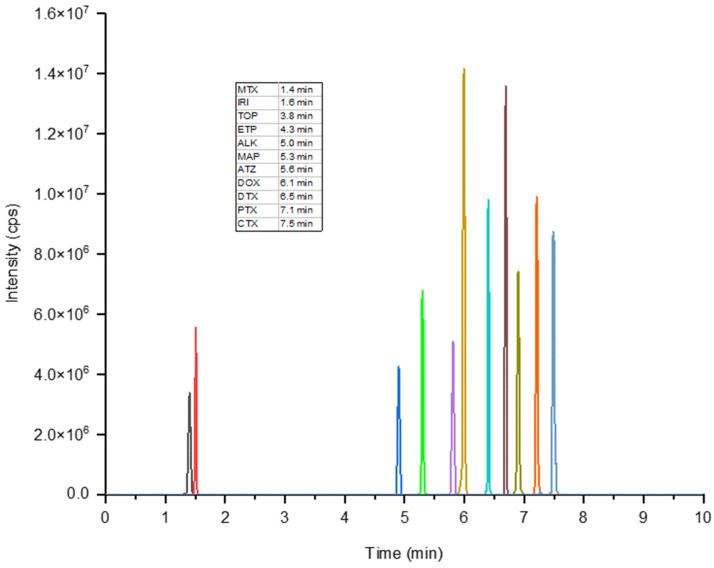
Representative chromatogram of the target pharmaceuticals in a mixture of standards (500 ng L^−1^). The peaks shown are for the quantifier ion transition of each target compound given in Table 2.

The optimized online SPE-LC-MS/MS method was validated in accordance with the ISO/IEC 17025 guideline. The parameters evaluated in the validation process were selectivity, linearity, LOD and LOQ, precision, and recovery. Figure 4 depicts a standard representative chromatogram obtained for a 500 ng L^−1^ mix of all target compounds, which contained a 250 ng L^−1^ of the IS, i.e., ATZ-d_5_.

The method’s selectivity was determined by comparing the MRM chromatograms of blank water samples with those obtained from the spiked ones. Considering the retention times of the analytes and IS, there were no peaks overlapping within the 1-min delta retention time window operated under dMRM mode (i.e., no interference), and satisfactory separation of all analytes was achieved. Table 3 summarizes the results of the validation procedure.

The linearity of the method was investigated by analyzing a calibration mix of standards at 11 concentration levels (LOQ, 1.00, 2.50, 5.00, 10.0, 25.0, 50.0, 100, 250, 500, 1.00 × 10^3^ ng L^−1^) prepared in methanol/water (10:90, *v*/*v*) in three independent replicates, which also contained 250 ng L^−1^ IS and 0.1% (*v*/*v*) formic acid. Due to the lack of isotope-labelled standards that could fit the set of pharmaceuticals targeted in this study, atrazine-d_5_ was used as the IS since good results were reported for multi-residue methods [26,63] containing four of the drugs targeted in this study. As can be seen in Table 3, all the calibration curves had good linearity with coefficients of determination (R^2^) greater than 0.99 for all compounds. The variances explained by the models were significant as confirmed by the F-test (*p* = 0.05) and no lack-of-fit was detected in any of them.

The limits of detection (LOD) and quantification (LOQ) were determined from the standard calibration curves using the Hubaux-Vos’s method [58]. LODs were all below 10 ng L^−1^ except for aliskiren which was only slightly higher (Table 3). In general, the LODs were in the ranges 1.30–10.6 ng L^−1^, while LOQs were in the range 4.30–35.5 ng L^−1^, indicating that the present method was highly sensitive allowing ultratrace quantification. In a method developed by Negreira et al. (2013), LODs slightly lower than those obtained in this study were reported for PTX (0.6 ng L^−1^), MTX (0.1 ng L^−1^), IRI (0.1 ng L^−1^), ETP (3.0 ng L^−1^) and DOX (0.1 ng L^−1^) using HPLC water and employing the PLRP-s online SPE cartridges [30]. These differences could be explained partly by the larger volume of sample (5 mL) loaded onto the online SPE against the 0.9 mL used in our study, and partly by the type SPE cartridge used.

One of the requirements for a well-established analytical method is the achievement of consistent and satisfactory results for precision and recovery analysis at varied concentration levels. Precision was evaluated by determining intraday and interday precisions, expressed as RSD (%). In all cases, the intraday precisions (*n* = 5) were below 8% and the interday RSD values (*n* = 15) fell below 13% (Table 3). In fact, intraday RSD (%) values were in the ranges 1.6–7.8 for QC_L_, 3.2–7.4 for QC_M_, and 2.1–6.7 for QC_H_. On the other hand, interday RSDs were in the ranges 7.0–13 for QC_L_, 4.3–9.4 for QC_M_, and 3.3–13 for QC_H_.

Recoveries obtained from spiked wastewater influent samples analyzed using the optimized method also ranged from 84.0% to 105.6% at QC_L_, 78.4% to 103.4% at QC_M_, and 79.9% to 111.0% at QC_H_ (Table 3). These values indicated that the method was reproducible for all the target analytes.

**Figure 5:**
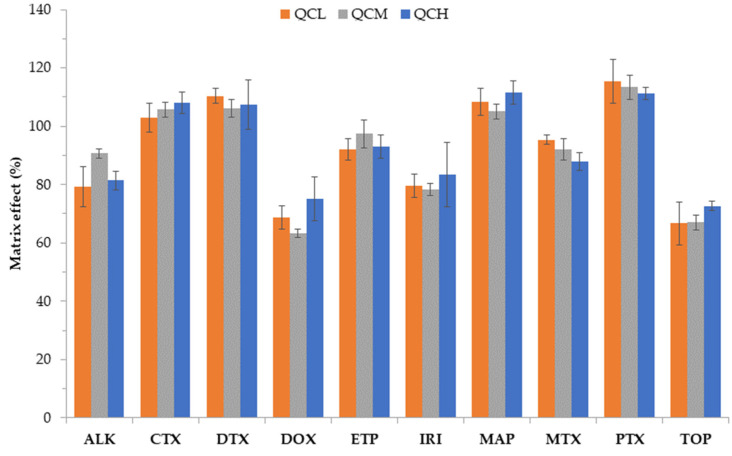
Matrix effects in wastewater influent samples.

Complex matrices can have a significant impact on target compound stability and extraction efficiency. The average matrix effects obtained in this study ranged from 69.0% to 113.0% (Figure 5). Signal suppression was observed for TOP (31%), DOX (31%), IRI (20%), ALK (16%), MTX (8%), ETP (6%), while signal enhancement was observed for CTX (6%), DTX (8%), MAP (8%), and PTX (13%). Some of these results agreed with previous studies which had reported ion suppression for IRI, ETP and DOX [26,30] in wastewater effluent and influents. Nevertheless, these effects did not influence quantification of the analytes.

### 3.4. Analysis of Real Water Samples

The developed method was applied to the analysis of six hospital wastewater effluents collected from Aalborg (Denmark) and Valencia (Spain), both of which use primary advanced treatment. During the analysis, both low- and high-level QCs spiked with the analytes at 100 ng L^−1^ and 800 ng L^−1^, respectively, were run in between samples. Potential carryover problems were evaluated with procedural blanks of pure HPLC water. Out of the ten target analytes, only MAP and MTX were detected respectively in 3 WWTP samples obtained from Denmark and 2 WWTP samples from Spain (see Table S5 for further details). The concentrations ranged from 11.2 to 23.1 ng L^−1^ for maprotiline and 4.7 to 9.3 ng L^−1^ for methotrexate.

In the literature, methotrexate has been reported as a widely consumed antineoplastic agent used for the treatment of acute lymphoblastic leukemia and its consumption in Spain, for example, has been estimated to be 144–196 g/day during the period 2010–2015 [31] with predicted environmental concentration (PEC) values of 1.5 and 0.056 ng L^−1^, respectively, in effluent and river waters. Previous studies have also reported the detection of methotrexate with concentrations of 12.6 ng L^−1^ in sewage treatment plant (STP) effluents from Italy [64], 1.6–18.1 ng L^−1^ in STP influents and up to 200 ng L^−1^ in hospital effluents [32], and 3.5–19.4 ng L^−1^ in WWTP influents [27]. Furthermore, maprotiline was previously reported at 0.4 ng L^−1^ in WWTP effluents [65] and up to 16.5 ng L^−1^ in EU WWTP effluents [66]. The other compounds were not detected in the hospital effluent samples, which may be explained by the fact that hospitals contribute a small proportion of pharmaceutical load as reported by Ort et al. [67] and Feldmann et al. [68], with over 85% of pharmaceutical loads considered in their studies found not to originate in hospitals. Similarly, another study [69] discovered that only 7.5% of antineoplastics included in their study were detected in hospital effluents, implying that most of the drugs detected were consumed by patients and excreted in household sewage.

## 4. Conclusions

A new rapid, sensitive, and fully automated online SPE–LC–MS/MS method has been developed and validated for the simultaneous determination of 10 multi-class pharmaceutical compounds in wastewater samples at ultratrace (ng L^−1^) levels. The Hypersil Gold SPE column was found to be the best preconcentration cartridge for all the analytes with the lowest average recoveries being for irinotecan (78.4%) and the highest for methotrexate (111.0%). The automation of the SPE procedure in tandem with the LC–MS/MS run resulted in analysis times per sample of only 10 min. Only two of the studied compounds (methotrexate and maprotiline) were found in relatively low quantities (between 4.70 and 23.1 ng L^−1^) in hospital effluents from Denmark and Spain. The methotrexate levels found in the present study were comparable with those previously found in hospital effluents in Italy and Spain but were far lower than those reported in China. However, the health consequences cannot be ignored. As a result, the sensitivity of the method is of the highest significance. To the best of the authors’ knowledge, this is the first multi-residue analytical method based on online SPE developed and validated for the determination of a group of pharmaceuticals including antineoplastic, antihypertensive, and antidepressant drugs. Furthermore, previously optimized methods for wastewater samples did not include maprotiline, aliskiren, cabazitaxel, docetaxel, and topotecan. Therefore, this new analytical method can be of great value to the evaluation of the target drugs in different wastewaters. The proposed method can be used to investigate the release of these emerging contaminants and, eventually, to examine the effectiveness of the wastewater treatment system in use. It is important to note that matrix effects were significant for certain compounds and the use of isotopically labeled internal standards is necessary for their accurate quantification in complex matrices.

## Figures and Tables

**Table 1:** Mobile phase program for the loading and analytical pumps (A = 0.1% (*v*/*v*) formic acid in water, B = methanol, C = 0.1% (*v*/*v*) formic acid in water, and D = 0.1% (*v*/*v*) formic acid in acetonitrile).

Time (min)	Loading Pump (SPE)			Valve Position	Analytical Pump (HPLC)	
	A (%)	B (%)	Flow Rate (mL min^−1^)		C (%)	D (%)
0.00	95.0	5.00	1.0	Loading	95.0	5.00
1.10	95.0	5.00	1.0	Loading	95.0	5.00
1.15	95.0	5.00	1.0	Injection	95.0	5.00
5.00	0.00	100	0.1	Injection	0.00	100
7.00	0.00	100	0.1	Injection	0.00	100
8.00	0.00	100	0.1	Injection	0.00	100
10.0	95.0	5.00	1.0	Loading	95.0	5.00
15.0	95.0	5.00	1.0	Loading	95.0	5.00

**Table 2:** Molecular formulas and the optimized LC-MS/MS parameters for analyzing the target pharmaceutical compounds. ATZ was used as the internal standard.

Compound (Abbreviation)	Formula	RT (min)	Precursor Ion, [M + H]^+^ (*m*/*z*)	Product Ions (*m/z*)	Frag (V)	CE (V)
Aliskiren (ALK)	C_30_H_53_N_3_O_6_	5.0	552.4	346.3; 534.5	135	27; 40
Cabazitaxel (CTX)	C_45_H_57_NO_14_	7.5	836.3	555.3; 433.1	135	20; 20
Docetaxel (DTX)	C_43_H_53_NO_14_	6.5	808.3	527.1; 509.0	135	20; 15
Doxorubicin (DOX)	C_27_H_29_NO_11_	6.1	544.0	361.2; 397.2	135	10; 10
Etoposide (ETP)	C_29_H_32_O_13_	4.3	589.2	229.1; 185.2	135	15; 15
Irinotecan (IRI)	C_33_H_38_N_4_O_6_	1.6	587.3	124.1; 167.1	120	45; 21
Maprotiline (MAP)	C_20_H_23_N	5.3	278.1	250.0; 191.1	135	15; 15
Methotrexate (MTX)	C_20_H_22_N_8_O_5_	1.4	455.2	308.1; 175.1	120	10; 25
Paclitaxel (PTX)	C_47_H_51_NO_14_	7.1	854.0	105.1; 286.0	100	19; 10
Topotecan (TOP)	C_23_H_23_N_3_O_5_	3.8	422.2	377.1; 320.0	120	10; 21
Atrazine-d_5_ (ATZ)	C_8_H_5_H_9_ClN_5_	5.6	221.1	179.2; 101.2	135	20; 20

**Table 3:** Method validation parameters (calibration range, linearity, LOD, LOQ, precision, and recovery). The lowest level of the calibration curve was always the LOQ value. Spiked QC levels for the evaluation of precision and recovery were QC_L_ (LOQ), QC_M_ (100 ng L^−1^) and QC_H_ (800 ng L^−1^) for each target compound. RSDs for recoveries shown in parentheses.

Compound	Linearity (R^2^)	LOD (ng L^−1^)	LOQ (ng L^−1^)	Spiked QC	Precision (RSD %)		Recovery (%)
					Intraday	Interday	
ALK	0.9978	10.7	35.5	QC_L_	5.6	13	101.6 (5.4)
				QC_M_	3.3	9.2	95.7 (10)
				QC_H_	3.7	7.9	94.6 (2.0)
CTX	0.9937	7.98	26.6	QC_L_	5.5	7.0	101.3 (2.2)
				QC_M_	6.0	4.3	94.7 (7.5)
				QC_H_	2.9	3.5	96.3 (7.1)
DTX	0.9987	2.67	8.89	QC_L_	7.8	11	84.0 (11)
				QC_M_	6.7	7.9	95.3 (8.9)
				QC_H_	8.4	6.4	96.9 (7.8)
DOX	0.9978	2.27	7.57	QC_L_	6.1	7.5	80 (13)
				QC_M_	4.1	9.1	85.8 (2.9)
				QC_H_	5.5	13	87.6 (9.1)
ETP	0.9947	3.25	10.9	QC_L_	3.7	9.6	104.5 (6.9)
				QC_M_	3.2	6.8	96.9 (3.8)
				QC_H_	6.2	12	93.3 (6.8)
IRI	0.9975	7.96	26.5	QC_L_	4.8	9.3	78.4 (7.6)
				QC_M_	5.1	8.0	89.2 (5.4)
				QC_H_	3.9	11	86.5 (4.4)
MAP	0.9997	1.30	4.34	QC_L_	2.6	8.5	103 (14)
				QC_M_	3.3	5.4	103.2 (6.4)
				QC_H_	2.1	5.5	98.6 (8.2)
MTX	0.9991	4.43	14.8	QC_L_	2.1	10	85.4 (9.0)
				QC_M_	4.4	7.7	94.4 (5.6)
				QC_H_	3.0	5.2	111.0 (6.4)
PTX	0.9969	6.99	23.3	QC_L_	6.6	7.8	96 (12)
				QC_M_	5.1	5.2	88.8 (9.2)
				QC_H_	6.7	3.3	94.6 (7.4)
TOP	0.9982	4.22	14.1	QC_L_	7.5	12	92 (11)
				QC_M_	7.4	9.4	93.7 (9.1)
				QC_H_	6.1	9.8	96.6 (7.4)

## Data Availability

The data that support the findings of this study are available from the corresponding author upon reasonable request.

## Supplementary Materials

The following supporting information can be downloaded at: https://www.mdpi.com/article/10.3390/toxics10030103/s1, Figure S1: CID product ions of doxorubicin and etoposide; Table S1: Formula, structure and logP values of the ten pharmaceutical compounds; Table S2: ESI source parameters used in the LC-MS/MS method; Table S3: Efficiency and asymmetry factor obtained using three different analytical columns; Table S4: Comparison of the recoveries obtained with four online SPE cartridges; Table S5: Analysis results of hospital effluent samples from Denmark and Spain; Optimizer S1: Optimization of compound-dependent MS parameters using MassHunter Optimizer.
