# Supplementary material for: A Fully Automated Online SPE-LC-MS/MS Method for the Determination of 10 Pharmaceuticals in Wastewater Samples
Source: Toxics. 2022 Feb 23;10(3):103. doi: 10.3390/toxics10030103 (PMC8955396; doi:10.3390/toxics10030103)
Supplement: Supplementary file 1 [file toxics-10-00103-s001.zip › toxics-1575701-supplementary.pdf]

toxics

MDPI

A Fully Automated Online SPE-LC-MS/MS Method for the Determination of 10 Pharmaceuticals in Wastewater Samples

Masho Hilawie Belay, Ulrich Precht, Peter Mortensen, Emilio Marengo and Elisa Robotti

Optimizer S1: Optimization of compound-dependent MS parameters using MassHunter Optimizer

The MRM method optimization was performed by injecting (with the column) 1.0 µg mL^{-1} individual standard solutions of the target compounds prepared in methanol/water (90:10, v/v), fine-tuning the fragmentor voltage from 100 to 200 V with a step of 5 V and the collision energy from 5 to 50 V with a step of 2 V, while the Cell Accelerator Voltage was fixed at 7 V. Mass spectral data was acquired both in electrospray ionization positive (ESI+) and negative (ESI-) polarity modes. Selection of the most abundant precursor ion, setting up a minimum abundance of 10000 counts, was made from $[M+H]^+$, $[M+NH_4]^+$, $[M+Na]^+$, and $[M+K]^+$ ions for positive ESI and $[M-H]^-$ for negative ESI, with a charge state of 1 in both cases. Product ion selection was performed with a low mass cut-off value of *m/z* 40 and excluding masses due to neutral losses of H_2O which had intensity of at least 1000 counts. Although both positive and negative ESI scans were performed, all target analytes were suitably ionized in positive ESI mode producing more abundant precursor and product ions compared to their counter parts in the negative ESI. The complete automatic optimization performed the selection of the best precursor ion, the optimization of the fragmentor voltage for each precursor ion, the selection of the best 4 product ions, and the optimization of the collision energy values for each transition for a list of the specified compounds. In the optimized MRM method, data acquisition was performed by recording the transitions between the precursor ion and the two most abundant product ions for each analyte. Each analyte had a unique quantifier ion (transition to the most abundant product ion) and a qualifier ion (transition to the second most abundant product ion).

It is important to note that results of the Optimizer for doxorubicin and etoposide were not well optimized (i.e., only one product ion fulfilling the 1000 counts abundance limit was detected). For both compounds, manual optimization was carried out which led to improved results (Figure S1). Thus, the optimal CE values were 10 V and 15 V respectively for DOX and ETP.

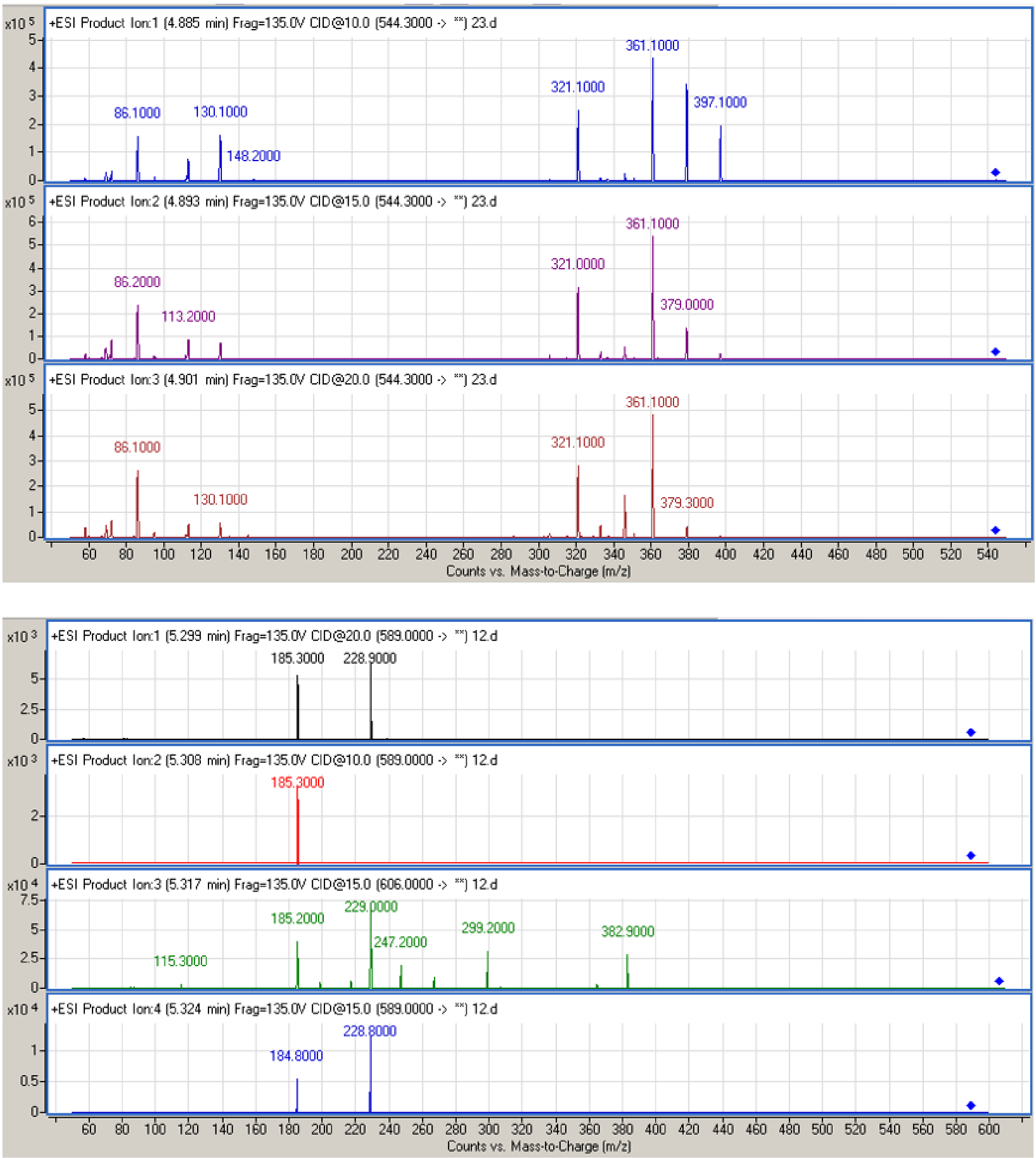

Figure S1. CID product ions of doxorubicin (top) and etoposide (bottom).

Table S1. Formula, structure and logP values of the ten pharmaceutical compounds

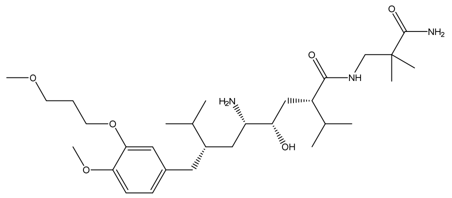 Aliskiren ($C_{30}H_{53}N_3O_6$); logP 3.51	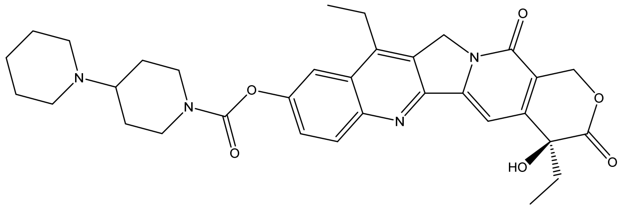 Irinotecan ($C_{33}H_{38}N_4O_6$); logP 2.43
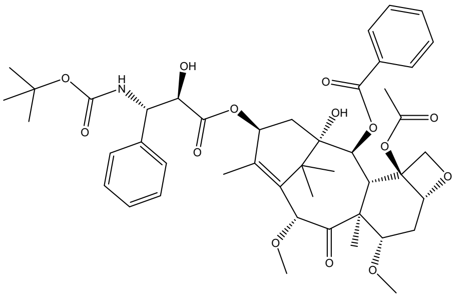 Cabazitaxel ($C_{45}H_{57}NO_{14}$); logP 5.44	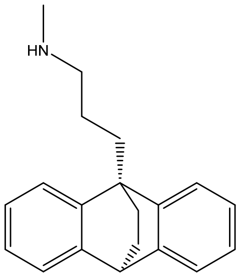 Maprotiline ($C_{20}H_{23}N$); logP 4.52
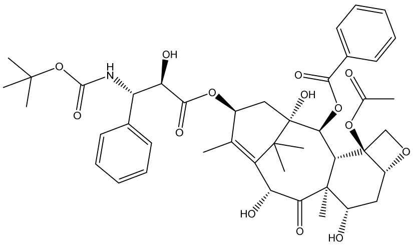 Docetaxel ($C_{43}H_{53}NO_{14}$); logP 4.08	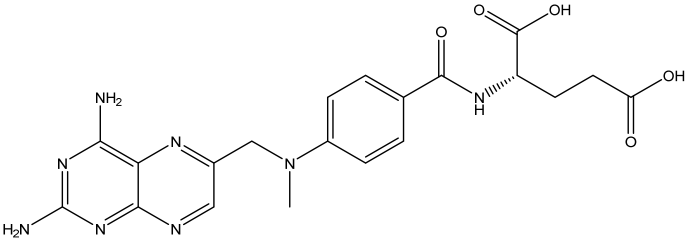 Methotrexate ($C_{20}H_{22}N_8O_5$); logP −0.53
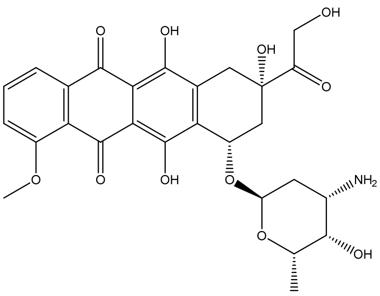 Doxorubicin ($C_{27}H_{29}NO_{11}$); logP 0.32	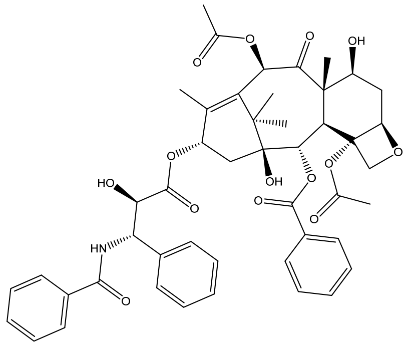 Paclitaxel ($C_{47}H_{51}NO_{14}$); logP 4.73
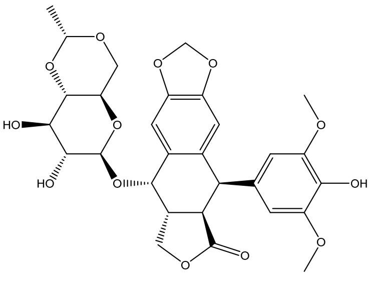 Etoposide ($C_{29}H_{32}O_{13}$); logP −0.11	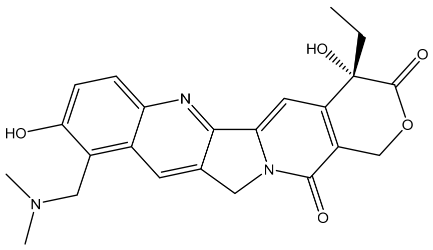 Topotecan ($C_{23}H_{23}N_3O_5$); logP 0.43

Table S2. Mass spectrometry parameters used in the LC-MS/MS method

Parameter	Value
Ionization mode	ESI Agilent JetStream
Polarity	Positive
Drying gas temperature	250 °C
Drying gas flow	8 L min^{-1}
Nebulizer pressure	45 psi
Sheath gas heater	350 °C
Sheath gas flow	11 L min^{-1}
Capillary voltage	2500 V
Vcharging	0
Delta electron multiplier voltage (EMV)	500
Scan type	Dynamic MRM

Table S3. Efficiency and asymmetry factor obtained using three different analytical columns (mean values obtained from triplicate analysis of three independent samples containing 1.0 µg/L mix of the ten analytes and the IS). Efficiency results are reported as: mean values*10^4 ± SD*10^2.

Compound	Efficiency (N)*			Asymmetry factor		
	Kinetex	Eclipse	Luna	Kinetex	Eclipse	Luna
Aliskiren (ALK)	8.4 ± 1.3	7.8 ± 2.2	6.7 ± 4.6	1.07 ± 0.05	1.07 ± 0.09	1.08 ± 0.15
Cabazitaxel (CTX)	6.1 ± 1.2	4.0 ± 6.9	4.0 ± 7.5	1.05 ± 0.16	1.16 ± 0.05	1.15 ± 0.06
Docetaxel (DTX)	4.9 ± 1.5	3.4 ± 3.6	9.1 ± 3.9	0.95 ± 0.07	1.22 ± 0.12	1.22 ± 0.13
Doxorubicin (DOX)	7.1 ± 1.2	6.4 ± 1.9	5.2 ± 5.0	1.57 ± 0.06	1.74 ± 0.04	1.74 ± 0.04
Etoposide (ETP)	7.4 ± 3.7	6.4 ± 5.8	5.3 ± 4.3	1.14 ± 0.19	1.66 ± 0.07	1.66 ± 0.07
Irinotecan (IRI)	6.2 ± 2.9	5.1 ± 4.9	5.1 ± 7.4	1.61 ± 0.08	1.88 ± 0.14	1.75 ± 0.14
Maprotiline (MAP)	6.5 ±1.0	5.7 ± 4.8	4.8 ± 1.4	1.08 ± 0.04	1.11 ± 0.07	1.22 ± 0.15
Methotrexate (MTX)	5.1 ± 2.4	4.8 ± 5.0	4.0 ± 8.1	1.17 ± 0.04	1.28 ± 0.19	1.20 ± 0.10
Paclitaxel (PTX)	9.6 ± 2.0	8.0 ± 3.2	7.7 ± 2.9	1.35 ± 0.09	1.48 ± 0.17	1.75±0.03
Topotecan (TOP)	5.8 ± 3.7	4.6 ± 2.6	4.4 ± 9.4	1.05 ± 0.09	1.17 ± 0.05	1.18 ± 0.19
Atrazine-d_5 (ATZ)	7.1 ± 1.3	7.2 ± 1.2	6.7 ± 1.9	0.98 ± 0.06	0.92 ± 0.09	1.28 ± 0.03

Table S4. Comparison of the recoveries obtained with four online SPE cartridges (analyte concentration 1.0 μg L^{-1}, sample volume 500 μL). Samples were analyzed in triplicate and individual percent recoveries were calculated as R_1, R_2, R_3. The average percentage recoveries ($R_{average}$) were also computed with the error estimates shown in parenthesis.

Compound	Oasis HLB				Hypersil Gold				PLPR-s				Hypercarb			
	R_1 (%)	R_2 (%)	R_3 (%)	$R_{average}$	R_1	R_2	R_3	$R_{average}$	R_1	R_2	R_3	$R_{average}$	R_1	R_2	R_3	$R_{average}$
TOP	78.0	64.2	64.2	68.8 (6.59	93.6	86.2	90.6	90.1 (3.0)	60.4	64.3	70.0	64.9 (3.9)	46.9	40.7	41.0	42.9 (2.9)
IRI	73.4	77.0	73.0	74.4 (1.8)	96.3	98.9	97.9	97.7 (1.1)	82.6	83.1	58.3	74.6 (11.6)	52.4	58.5	50.3	53.7 (3.5)
DOX	37.4	31.9	35.4	34.9 (2.3)	56.0	55.3	55.0	55.4 (0.4)	24.7	25.0	25.0	24.9 (0.1)	17.8	23.2	18.9	20.0 (2.4)
MAP	70.0	71.1	69.4	70.2 (0.7)	94.5	94.9	94.4	94.6 (0.2)	75.4	79.5	76.2	77.0 (1.8)	53.5	57.5	52.4	54.5 (2.2)
PTX	82.6	76.1	72.5	77.1 (4.2)	69.5	69.1	69.5	69.4 (0.2)	62.9	62.6	60.7	62.1 (1.0)	40.4	50.0	34.8	41.7 (6.3)
ALK	68.2	74.7	61.5	68.1 (5.4)	90.0	92.4	91.0	91.1 (1.0)	63.4	62.1	62.2	62.5 (0.6)	49.9	25.1	22.3	32.4 (12.4)
ETP	48.3	61.7	50.5	53.5 (5.9)	52.9	54.7	53.4	53.7 (0.8)	62.2	60.8	72.1	65.1 (5.0)	13.9	43.5	23.8	27.1 (12.3)
MTX	74.0	60.6	50.7	61.8 (9.6)	68.1	69.9	67.1	68.4 (1.2)	43.9	52.7	48.4	48.3 (3.6)	34.6	33.6	33.8	34.0 (0.4)
DTX	70.8	72.8	73.6	72.4 (1.1)	92.9	92.0	91.8	92.2 (0.4)	92.3	95.1	95.1	94.2 (1.3)	67.6	64.9	65.3	65.9 (1.2)
CTX	63.2	65.9	70.1	66.4 (2.8)	88.6	89.9	88.7	89.1 (0.6)	72.8	72.3	72.1	72.4 (0.3)	42.1	50.8	45.8	46.3 (3.6)

Table S5. Analysis results of hospital effluent samples from Denmark (A1 and A2) and Spain (V1-V4). <LOD = below limit of detection.

**Code**	**Collection date**	**Concentration (ng L^{-1})**									
		**ALK**	**CTX**	**DTX**	**DOX**	**ETP**	**IRI**	**MAP**	**MTX**	**PTX**	**TOP**
**A1**	12/02/2020	<LOD	<LOD	<LOD	<LOD	<LOD	<LOD	<LOD	**4.7**	<LOD	<LOD
**A2**	20/02/2020	<LOD	<LOD	<LOD	<LOD	<LOD	<LOD	<LOD	**9.3**	<LOD	<LOD
**V1**	19/02/2020	<LOD	<LOD	<LOD	<LOD	<LOD	<LOD	**23.1**	<LOD	<LOD	<LOD
**V2**	19/02/2020	<LOD	<LOD	<LOD	<LOD	<LOD	<LOD	**11.2**	<LOD	<LOD	<LOD
**V3**	19/02/2020	<LOD	<LOD	<LOD	<LOD	<LOD	<LOD	<LOD	<LOD	<LOD	<LOD
**V4**	19/02/2020	<LOD	<LOD	<LOD	<LOD	<LOD	<LOD	**20.2**	<LOD	<LOD	<LOD
